# Polysomnographic Characteristics of Sleep in Stroke: A Systematic Review and Meta-Analysis

**DOI:** 10.1371/journal.pone.0148496

**Published:** 2016-03-07

**Authors:** Chiara Baglioni, Christoph Nissen, Adrian Schweinoch, Dieter Riemann, Kai Spiegelhalder, Mathias Berger, Cornelius Weiller, Annette Sterr

**Affiliations:** 1 Department of Psychiatry and Psychotherapy, University Medical Center Freiburg, Freiburg, Germany; 2 Department of Neurology, University Medical Center Freiburg, Freiburg, Germany; 3 School of Psychology, University of Surrey, Surrey, United Kingdom; 4 Department of Neurology, University of Sao Paulo, Sao Paulo, Brazil; University of Rome Tor Vergata, ITALY

## Abstract

**Background:**

Research on sleep after stroke has focused mainly on sleep disordered breathing. However, the extend to which sleep physiology is altered in stroke survivors, how these alterations compare to healthy volunteers, and how sleep changes might affect recovery as well as physical and mental health has yet to be fully researched. Motivated by the view that a deeper understanding of sleep in stroke is needed to account for its role in health and well-being as well as its relevance for recovery and rehabilitation, we conducted a systematic review and meta-analysis of polysomnographic studies comparing stroke to control populations.

**Method:**

Medline and PsycInfo databases were searched using "stroke" and words capturing polysomnographic parameters as search terms. This yielded 1692 abstracts for screening, with 15 meeting the criteria for systematic review and 9 for meta-analysis. Prisma best practice guidelines were followed for the systematic review; the Comprehensive Meta-Analysis software was used for random effects modelling.

**Results:**

The meta-analysis revealed that patients with stroke have poorer sleep than controls. Patients had lower sleep efficiency (mean 75% vs 84%), shorter total-sleep-time (309.4 vs 340.3 min) and more wake-after-sleep-onset (97.2 vs 53.8 min). Patients also spend more time in stage 1 (13% vs 10%) and less time in stage 2 sleep (36% vs 45%) and slow-wave-sleep (10% vs 12%). No group differences were identified for REM sleep. The systematic review revealed a strong bias towards studies in the early recovery phase of stroke, with no study reporting specifically on patients in the chronic state. Moreover, participants in the control groups included community samples as well as other patients groups.

**Conclusions:**

These results indicate poorer sleep in patients with stroke than controls. While strongly suggestive in nature, the evidence base is limited and methodologically diverse, and hands a clear mandate for further research. A particular need regards polysomnographic studies in chronic community-dwelling patients compared to age-matched individuals.

## Introduction

Stroke is a major public health problem across the world and huge efforts are made to improve the long-term prospects for patients. However, as major potential contributor to stroke outcome, sleep is presently not fully considered. For example the recently revised guideline for stroke rehabilitation issued by the UK National Institute of Clinical Excellent (NICE [1] provides a detailed account of the medical, physical and psychological needs to be met through in-and outpatients stroke care, but these guidelines make no comment on sleep.

At the same time, sleep is known to be critical for physical health, quality of life and overall well-being in diseased as well as non-diseased populations (e.g. [2–5]). Initial evidence further suggests that motor learning after stroke can be facilitated by sleep [6]. Moreover, slow wave sleep increases following a session of intensive imitation-based speech and language therapy for aphasia, providing support for the idea that sleep and treatment-induced rehabilitation might be linked [7]. Studies in healthy controls further demonstrate the negative impact of poor sleep on daytime function [8–10], an effect which is most likely aggravated in stroke survivors with cognitive and/or physical impairment. Together with the substantive body of literature showing a strong association between sleep disordered breathing and stroke (for review see [11,12], these findings all point towards an important role of sleep in patients with stroke. However, at present, sleep is rarely considered in in-patient and community-based stroke care. This is despite a number of studies using subjective measures of sleep showing that patients with stroke often experience difficulties with their sleep (e.g. [13–15]).

In this paper, we argue that sleep is relevant for a patient’s ability to achieve their full potential for recovery and to live a fulfilled life post-stroke. A deeper and more comprehensive understanding of sleep, derived from objective polysomnographic (PSG) measures, is therefore required. Moreover, sleep characteristics observed in stroke need to be contextualised by the evidence-base on sleep and sleep disorders in the general population, in order to fully capitalise on the theoretical and clinical knowledge available. At present no review summarizes the key characteristics of sleep physiology after stroke. Simple questions, such as ‘is sleep architecture in stroke different from the characteristics typical for the respective age group’ are presently not fully answered. We therefore conducted a systematic review and meta-analysis of the literature reporting PSG recordings in these patients, and in comparison to control populations.

The focus on PSG was chosen for three reasons. Firstly, this methodology represents the current gold standard for sleep assessment. Secondly, this method affords a detailed examination of sleep continuity as well as sleep architecture. Thirdly, PSG is the best method for diagnosing organic sleep disorders, such as sleep disordered breathing and periodic limb movement disorder, and the only method to reliably determine the physiological causes of poor sleep. For the present review we determine markers of sleep continuity and sleep architecture, and further analysed parameters of organic sleep disorders and daytime function. All parameters were compared to control populations in order to determine how sleep changes in patients with stroke deviate from the sleep characteristics typical for persons without stroke.

## Method

### Best practice statement

The Preferred Reporting Items for Systematic Reviews and Meta-analyses (PRISMA[16]) guidelines were applied for the selection of suitable studies (for PRISMA checklist see S1 PRISMA Checklist). C.B. and A.Sc. independently conducted the search and screened titles and abstracts. For data extraction, full texts were examined by A.St., A.Sc., and C.B.

### Search method and study selection

A literature search for PSG studies in people with stroke published in the databases Medline and PsycInfo between January 1980 to July 2014 was performed using the search terms ‘stroke’ and ‘sleep’ or ‘polysomnogr*’ or ‘PSG’ or ‘PLMS’ or ‘RLS’ or ‘sleepiness’ or ‘Epworth sleepiness scale’ or ‘Stanford sleepiness scale’ or ‘Karolinska sleepiness scale’ or ‘MSLT’ or ‘MWT’. The terms were searched in the Title/Abstract or Keywords section. Selection criteria comprised: 1) PSG: only studies reporting a minimum of one PSG night were included; studies reporting measures of sleep apnea or daytime sleepiness only were excluded; 2) language: written in English, German, Italian, Spanish or French; 3) type of study: original research in humans (excluded reviews, comments, editorial, single-case studies); 4) control group: only studies comparing PSG data from stroke patients with either healthy or patient control groups were included; studies reporting repeated measures data in stroke only (i.e. no control group) were excluded. Further studies were added by examining the reference lists of identified papers. Unpublished studies were not included in order to focus on those with the most rigorous research methodology subject to peer review. We further excluded all studies presenting data from patients with transient ischemic attack (TIA). This meant that one of the largest and best-controlled studies [17], reporting data from 86 TIA patients and 86 healthy controls, was not considered.

### Data extraction

Two groups of variables were extracted. General characteristics comprising number of participants, age, gender, type of stroke, chronicity (time since stroke), control group characteristics (healthy-control participants vs hospitalized-control populations), medication, type of recruitment (in a clinic or in the general population) and number of PSG nights. Sleep characteristics comprised: 1) sleep continuity (total sleep time, TST; sleep efficiency index, SEI; sleep onset latency, SOL; wake after sleep onset, WASO; number of awakenings, NA); 2) sleep architecture (duration of stage 1 and 2 sleep; slow-wave sleep, SWS; rapid eye movement sleep, REM sleep; REM sleep latency, REML; REM sleep density, REMD); 3) other sleep parameters (arousal index, AI; sleep spindles; apnea-hypopnea index, AHI; periodic limb movement index, PLMI); and 4) daytime sleepiness: Epworth sleepiness scale (ESS) and multiple sleep latency test (MSLT). The definitions of these sleep variables are given in S1 Table.

Note that some of these sleep parameters can be calculated in more than one way. For example, the duration of the sleep stages can be reported in minutes or as the percentage of the time spent in bed or the total sleep time. These different modes of calculation imply that the respective data could not be collapse for meta-analysis, and separate analyses were therefore conducted in these situations.

Other relevant sleep variables we intended to capture were not measured in any of the studies meeting the quality criteria. These variables comprised: ‘restless leg syndrome- RLS’, ‘Stanford sleepiness scale’, ‘Karolinska sleepiness scale’, and ‘wake maintenance test’.

### Specific methods for the systematic review

The studies selected for systematic review were assessed for quality using the principles of the critical appraisal skills program for case-control studies tool (http://www.casp-uk.net/) as reported in Table 1. This instrument is designed for the judgement of study quality in a systematic and transparent way. The quality judgement is thereby derived through a set of standardized methodological questions applied to all studies under consideration. For the aims of the present study, the questions chosen for the quality assessment focused on the following themes: research question; recruitment; control group characteristics; stringency of sleep measures; and confounding factors.

**Table 1:** Assessment of the quality of the studies.

**Studies included in the meta-analysis**						
Study	Was the research question stated clearly?	Were the patients with stroke recruited in an acceptable way?	Is the control group a non-clinical population?	Is the control group adequately described?	Was sleep assessed and reported comprehensively?*	What confounding variables have the authors accounted for?
Arzt et al. 2010 [21]	Yes	Yes	Yes	Yes^1^	Yes	BMI (m ± sd)
Terzoudi et al. 2009 [26]	Yes	Yes	No	Yes	Yes	Barthel Index, m score
Gottselig et al. 2002 [27]	Yes	Yes	No	Yes	Yes	/
Müller et al. 2002 [28]	Yes	Yes	No	Yes	Yes	/
Bassetti and Aldrich 2001 [29]	Yes	Yes	No	Yes^1^	Yes	Scandinavian Stroke Scale (m ± sd)
Santamaria et al. 2000 [30]	Yes	Yes	Yes	Yes	Yes	n for hypertension, diabetes, smoking
Mohsenin and Valor 1995 [33]	Yes	Yes	No	No	Yes	BMI (m ± sd); hypertension %; smoking; snoring %; cardiovascular disorder
Hudgel et al. 1993 [34]	Yes	Yes	Yes	No	Yes	BMI can be calculated
Giubilei et al. 1992 [32]	Yes	Yes	No	No	Yes	/
**Studies excluded in the meta-analysis (but included in systematic review)**						
Coelho et al. 2010 [22]	Yes	No	No	No	Yes	hypertension %; polyneuropathy %; anemia %; antidepressants %; fatigue %
Siccoli et al. 2008 [25]	Yes	Yes	No	No	No^3^	m and range for BDI
Bliwise et al. 2002 [23]	Yes	Yes	No	Yes^2^	Yes	/
Vock et al. 2002 [31]	Yes	Yes	No	Yes	No^3^	/
Pinto et al. 2000 [35]	Yes	Yes	Yes	Yes	No^3^	/
Yokoyama et al. 1996 [24]	Yes	Yes	Yes	No	No^4^	/

* For comprehensively is meant that the study reported means with all data necessary to conduct meta-analytic calculations. 1 = no information about clinical assessment reported. 2 = control group characterized but comprising two groups of patients with Alzheimer and Parkinson diseases. 3 = not possible to calculate the effect size. 4 = sleep variables not considered in other studies, thus not possible to conduct meta-analytic calculations for only one study.

### Specific methods for the meta-analysis

All meta-analytic calculations were performed with the software ‘Comprehensive Meta-Analysis’, version 2[18]. For each sleep variable, defined in Table 1, a separate meta-analysis was conducted. Effect sizes were calculated as standardized mean (Cohen’s d). This implied that only studies reporting means and standard deviations could be included. For those studies not reporting this information but otherwise meeting the selection criteria, authors were contacted to provide the respective data. If the information was received, those studies could be included in the meta-analysis as well.

The random-effects model was used for data pooling (chi-squared test). The I^2^ statistic, derived from the chi-squared values, was used to determine heterogeneity, with low heterogeneity defined as I^2^ = 25%, moderate heterogeneity I^2^ = 50%, and high heterogeneity as I^2^ = 75%. An I^2^ close to 0 was considered to reflect primarily random error.

For outlier identification, we followed the standardized-residuals method suggested by Hedges and Olkin[19]. This method proclaims to exclude studies with standardized residuals greater or equal to +3 and/or lower or equal to -3. Based on this criterion, no study was excluded as outlier. Moreover, testing for publication bias is recommended when there are 10 or more trials pooled for a given analysis because the power with fewer studies is too low to distinguish chance from true publication bias[20]. Because none of our individual analyses included 10 or more trials, we did not evaluate publication bias.

Because of the small number of studies available for meta-analysis, and the heterogeneity of the methods used in those studies, potentially influential factors such as time since stroke or type of control group could not be fully considered. However, recognizing this caveat while also recognizing the challenge in performing PSG studies in patients, we conducted exploratory analysis for these two parameters and report this data separately as ‘subgroup analysis’ in the results section. For other key factors, such as age, sex, stroke type, medication, type of recruitment (clinic vs community), or number of nights of PSG, the data reported in the literature was too limited to allow any exploratory analysis. This in itself highlights the need for future research.

## Results

### Systematic Review (15 studies)

**Fig 1:**
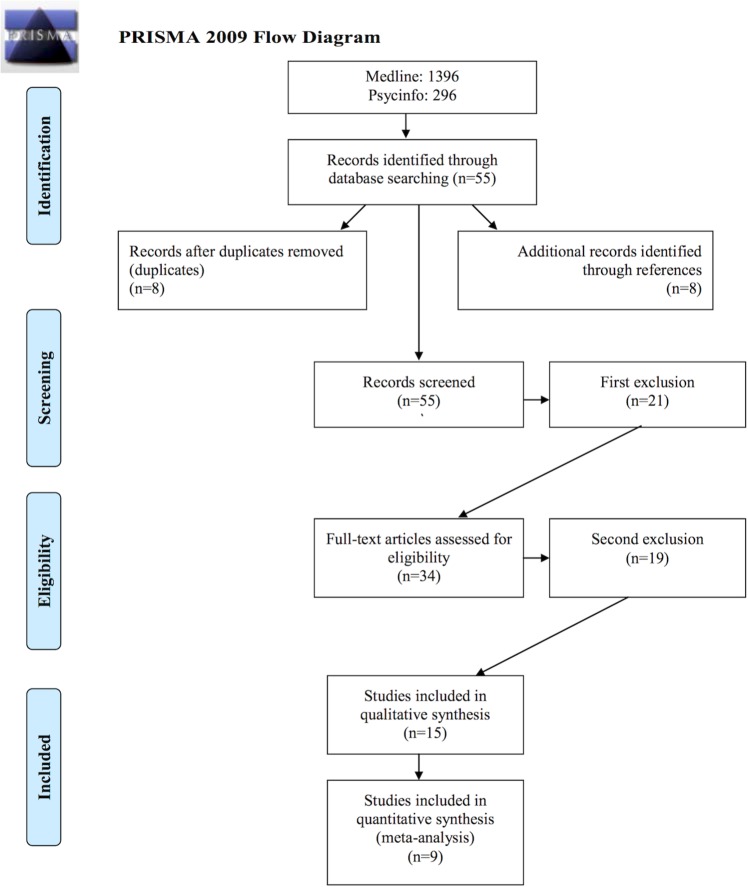
Prisma chart for search flow.

Fig 1 depicts the search flow for the 1692 abstracts identified. Applying the criteria specified in the method section, 1637 abstracts were excluded for the following reasons: 667 type of publication (e.g. reviews); 102 language; 46 animal studies; 605 neither stroke nor control group; 217 no PSG.

The full-text examination of the remaining 55 studies leaded to the exclusion of further 40 articles. These studies, and the reasons for their exclusion, are presented in S2 Table. In brief, 25 studies had no control group, 11 studies only reported breathing-related parameters but not PSG, one study reported the averaged data from various neurological conditions rather than the stroke group per se, one study was single case, one study focused on patients with TIA, and another on traumatic brain injuries.

The characteristics of the 15 papers remaining in the systematic review are detailed in Table 2. Across these 15 studies, 234 patients with stroke were compared to 169 hospitalized control participants; and 176 patients were compared to 1154 non-hospitalized healthy controls. The studies covered an age-range of 17 to 84 years of age, with 4 studies reporting no age range [21–24]. All studies reported the mean age and standard deviations or standard errors, with the exception of Siccoli et al [25], which provided age information for patients, but not for controls. All studies included both genders, and recruited patients through the clinic. No study reported sleep parameters for males and females separately. Moreover, several studies did not match the groups for gender. With regard to chronicity (time since stroke), 7 [21,23,26–30] out of the 15 studies examined sleep in the first 16 days post stroke. In one study, chronicity was not reported [22]. The remaining studies covered a diverse range of chronicity often within the same study, with one study collating the data acquired between 8 days and 1 year [25] post stroke; one for 8 to 35 days [31] one for 2 days to 3 weeks [32]; one for 3 months to 1 year [33]; and 3 for chronicities greater than 1 month [24,34,35]. We further noted that only 4 studies [22,23,27,31] specified the medication status of patients and controls, while 9 [21,24,25,28–30,33–35] did not report this type of information at all. Another 2 [26,32] were conducted in participants free of medication for the study period. Except for 2 studies [30,32], PSG was conducted on one single night only. In all studies the patient group comprised mixed stroke with regards to etiology and lesion location. This information is summarized for each study in Table 2.

**Table 2:** Study characteristics.

**Studies included in the meta-analysis**											
**Study**	**Stroke Group (N)**	**Other Group (which and N)**	**Control Group (N)**	**Control Group (type)**	**Age group**	**Sex**	**Stroke type**	**Chronicity**	**Medication**	**Recruitment**	**PSG night**
**Arzt et al 2010 [21]**	96	/	1093	Community	Mixed adults/elderly (m ± sd)	MX	Embolic, thromboembolic or hemorrhagic strokes	Time of admission	Not reported	1 = clinic	1 night
**Terzoudi et al 2009 [26]**	58	/	16	Patients with no major medical or psychiatric disorder	Mixed adults/ elderly (18–82 yrs)	MX	Ischaemich or haemorrhagic	Range: 6–10 days	Free of medication	1 = clinic	1 night
**Gottselig et al 2002 [27]**	30	/	12	Patients with peripheral neurological disease	Mixed young adults, adults and elderly (17–75)	MX	Unilateral hemispheric stroke	Within first 10 days	On medication	1 = clinic	1 night
**Müller et al 2002 [28]**	20	/	10	Patients hospitalized in the neurology (9) or dermatology (1) wards	Mixed adults/ elderly (18–80 yrs)	MX	First acute hemispheric stroke	Within 8 days	Not reported	1 = clinic	1 night
**Bassetti and Aldrich 2001 [29]**	24	TIA: 17	TIA group	TIA	Mixed adults/ elderly (26–78 yrs)	MX	Supratentorialextrathalamic stroke	M 11.7 days	Not reported	1 = clinic	1 night
**Santamaria et al 2000 [30]**	13	/	18	Healthy volunteers	Mixed adults/ elderly (37–84 yrs)	MX	Acute unilateral thalamic stroke	M 14 days (range: 7–21)	Not reported	1 = clinic	2 nights (1st as adaptation)
**Mohsenin and Valor 1995 [33]**	10	/	10	Other patients	Mixed adults/ elderly (27–78 yrs)	MX	Cerebral hemispheres without brainstem lesion	M 3 mo(within 1year)	Not reported	1 = clinic	1 night
**Hudgel et al 1993 [34]**	8	/	8	Healthy volunteers	Elderly (> 65 yrs)	MX	Unilateral cerebral hemorrhagic or ischemic stroke	> or = 1 mo	Not reported	1 = clinic	1 night
**Giubilei et al 1992 [32]**	17	17 (repeated measures)	10	Patients with peripheral neurological disease	Mixed adults/ elderly (47–79 yrs)	MX	Ischemic stroke in MCA	2nd night and 3 weeks later	Free of medication	1 = clinic	2 or 3 nights(1st night as adaptation)
**Studies excluded in the meta-analysis (but included in systematic review)**											
**Study**	**Stroke Group (N)**	**Other Group (which and N)**	**Control Group (N)**	**Control Group (type)**	**Age group**	**Sex**	**Stroke type**	**Chronicity**	**Medication**	**Recruitment**	**PSG night**
**Coelho et al 2010 [22]**	40	/	40	Patients of the sleep lab	Mixed adults/elderly (m ± sd)	MX	Not specified	Not reported	On medication	1 = sleep lab	1 night
**Siccoli et al 2008 [25]**	11	9 (repeated measures)	5	Patients with no neurological or psychiatric disorder	Working age adults (18–59 yrs)	MX	Acute ischaemic hemispheric stroke	Within 8 days and between 3 and 12 mo at discharge	Not reported	1 = clinic	1 night
**Bliwise et al 2002 [23]**	9	Alzheimer: 6	Parkinson:32	Patients with other neurological disorder	Mixed adults/elderly (m ± sd)	MX	Subcortical	M 16 days	On medication	1 = clinic	1 night
**Vock et al 2002 [31]**	15	15 (repeated measures)	11	Patients with neurological (10) or dermatological disorder (1)	Mixed adults/ elderly (18–76 yrs)	MX	Hemispheric ischemic stroke	Within 8 to 35 days	On medication	1 = clinic	at least once
**Pinto et al. 2000 [35]**	24	/	24	Healthy volunterers	Mixed adults/elderly (32–69 yrs)	MX	Vascular stroke (mixed)	> 1 mo	Not reported	1 = clinic	1 night
**Yokoyama et al 1996 [24]**	35	/	11	Normal subjects	Mixed adults/elderly (m ± sd)	MX	Unilateral supra-tentorial lesions	> 2 mo	Not reported	1 = clinic	1 night

With regards to OSA, five studies reported AHI values or presence/absence of OSA. In one of these studies [26], 34 of 58 patients had sleep disordered breathing, but no AHI values were reported. Another study mentioned that 54% of participating patients had AHI values > 10^(29)^, while Coelho et al. [22] specified that 65% of the patient sample had AHI values > 5. In a further study [33], 9 of 10 patients were diagnosed with OSA. Finally, Pinto et al. [35] reported that 6 of 24 patients presented respiratory alterations with indexes ranging from 10.1 to 59.4/hour of sleep. Four studies reported no information on AHI values nor OSA diagnosis [23–25,30]. Three studies specified that all patients had AHI values < 10 [27,28,31] and one study reported that patients had no history of sleep disorders [32]. Hudgel et al. [34] controlled for presence/absence of OSA, but no detailed information is given in the manuscript. Finally, Arzt et al. [21] included three categories of patients and controls based on AHI index values. No OSA was defined as AHI <5 (patients N = 46), mild OSA as AHI between 5–15 (patients N = 32), and moderate to severe OSA as AHI > 15 (patients N = 18). Moreover, 13 of 15 studies reported no information on daytime sleepiness [22–30,32–35]. One study [21] used the Epworth Sleepiness Scale (ESS) to assess daytime sleepiness and found that patients with stroke presented with significantly lower ESS scores than participants from the community in all OSA categories. Finally, Vock et al. [31] reported for each patient the ESS score before and after stroke. In addition, the index of arousal was not reported in any study. Thirteen of 15 studies reported no desaturation index [21,23–33,35]. Hudgel et al. [34] reported respiratory variables values for each patient. Coelho et al. [22] specify that patients and controls did not differ in percentage of lowest oxygen.

### Meta-analysis (9 studies)

Quality assessment showed that 9 studies had sufficient information to be used for meta-analytic calculations (Table 1). There were 6 studies included in the systematic review which were not included in the meta-analysis. Three [25,31,35] of these 6 studies could not be included because they lacked the information required for effect sizes calculation. Another study [22] relied entirely on self-report for stroke diagnosis and did not include information on either location, time after stroke, initial severity or long-term outcome. The control population in the study by Bliwise et al [23] comprised patients with Alzheimer and Parkinson diseases, which rendered effect sizes comparisons none sensible. The study by Yokoyama et al. [24] analysed sleep-related EEG spectral analyses which were not reported in any other study; thus no meta-analysis was possible.

**Fig 2:**
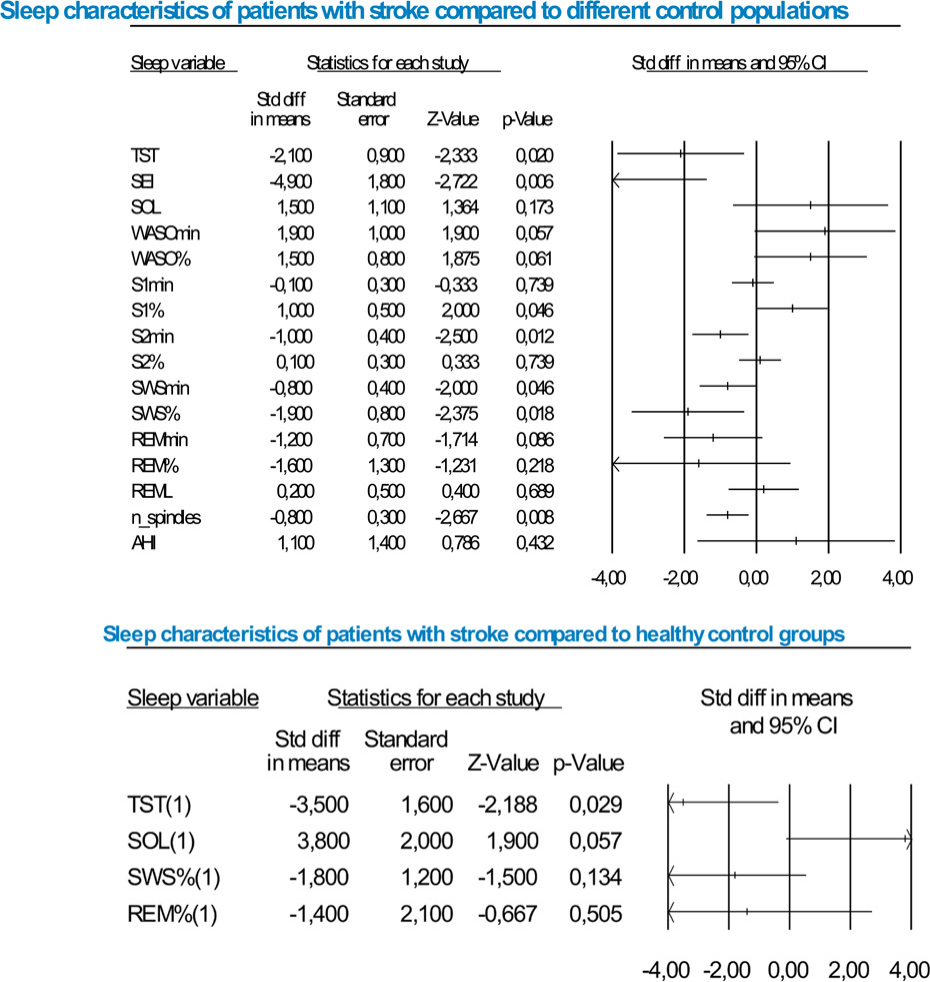
Graphical representation of meta-analysis data. Top half shows the results for all studies (with hospitalized and non-hopitalized control groups). The bottom half shows the results for studies with non-hospitalized healthy control groups.

Because of the limited number of studies available, the minimum of studies needed to calculate a meta-analysis was set to 2. This cut-off criterion was applied to all sleep variables. Despite this low criterion, no meta-analysis could be calculated for REMD, PLMI, MSLT and AI. The mean values for each sleep variable together with the number of studies available for effect size statistics are presented in Table 3, and listed separately for the studies included in the systematic review and for those included in the meta-analysis. A graphical illustration of the results is presented in Fig 2. In the section below, the results are detailed for each sleep variable. No study had to be excluded as an outlier.

**Table 3:** Mean values of each sleep variable for each study.

**Studies included in the meta-analysis**														
**Study**	** **		**Arzt et al 2010 [21]: AHI<5**	**Arzt et al 2010 [21]: 5≤AHI<15**	**Arzt et al 2010 [21]: AHI≥15**	**Terzoudi et al 2009 [26]**	**Gottselig et al 2002 [27]**	**Müller et al 2002 [28]**	**Bassetti and Aldrich 2001[29]**	**Santamaria et al 2000 [30]**	**Mohesenin and Valor 1995 [33]**	**Hudgel et al 1993 [34]**	**Giubilei et al 1992 [32]_ ACUTE**	**Giubilei et al 1992 [32]_ AFTER 3 WEEKS**
**Sleep continuity**														
**TST** SG	10 studies		354,0±12,0	367,0±11,0	322,0±17,0	242,4±93,6	402,6±17,1	388,0±75,0	246,2±14,6	243,2±75,9	227,0±35,0	100,0±12,0	416,8±128,8	403,9±96,6
**TST** CG	10 studies		384,0±2,0	382,0±5,0	359,0±7,0	299,0±51,1	425,9±11,2	416,0±48,0	304,0±10,9	334,7±65,7	271,0±26,0	113,0±16,0	397,5±68,7	397,5±68,7
**SEI** SG	6 studies		76,0±2,0	76,9±2,1	70,5±3,1	75,3±17,8	78,7±2,6	76,0±10,0	69,1±2,7					
**SEI** CG	6 studies		84,3±0,4	83,6±0,9	79,5±1,3	87,6±7,9	88,3±2,4	86,0±8,0	78,4±2,5					
**SOL** SG	7 studies		20,7±3,0	16,6±3,6	25,6±7,4	42,2±42,5	21,7± 2,6	33,0±31,0	37,7±11,1	21,40±28,9	13,0±5,0			
**SOL** CG	7 studies		13,0±0,6	13,2±2,0	11,7±1,1	27,2±11,2	26,7±4,7	28,0±17,0	29,5± 6,4	16,4±14,5	26,0±4,0			
**WASOmin** SG	3 studies						105,5±13,6	112,0±53,0		74,2±48,3				
**WASOmin** CG	3 studies						50,4±12,0	60,0±38,0		51,1± 35,0				
**WASO%** SG	2 studies					24,8±17,8					42,0±9,0			
**WASO%** SG	2 studies					12,4±7,8					26,0±4,0			
**NA** SG1	1 study												31,3±16,7	17,8±9,9
**NA** SG1	1 study												8,1±2,5	8,1±2,5
**Sleep architecture**														
**S1min** SG		2 studies					60,1±6,4	66,0±31,0						
**S1min** CG		2 studies					62,7±8,0	58,0±24,0						
**S1%** SG		4 studies				7,2±7,3			23,2±2,7	9,5± 5,9	12,0±3,0			
**S1%** CG		4 studies				6,6±2,2			18,7±2,0	8,0± 4,9	8,0±1,0			
**S2min** SG		2 studies					246,9±14,6	223,0±60,0						
**S2min** CG		2 studies					243,0±11,2	232,0±28,0						
**S2%** SG		4 studies				53,2±18,3			25,1±3,2	29,3± 14,4	36,0±6,0			
**S2%** CG		4 studies				58,0±4,7			34,9±9,2	41,6± 19,2	45,0±3,0			
**SWSmin** SG		2 studies					31,4±5,0	34,0±32,0						
**SWSmin** CG		2 studies					39,1±9,3	45,0±34,0						
**SWS%** SG		5 studies	12,0±1,4	10,5±1,5	10,9±2,1	6,4±5,8			1,6±0,5	24,2± 16,1	4,0±2,0			
**SWS%** CG		5 studies	14,5±0,4	12,4±0,7	11,2±1,0	10,4±2,5			5,2±1,8	19,5± 12,5	9,0±2,0			
**REMmin** SG		3 studies					64,2±6,0	66,0±35,0					8,3±16,3	71,2±25,0
**REMmin** CG		3 studies					81,1±6,0	82,0±210					68,5±36,6	68,5±36,6
**REM%** SG		5 studies	16,9±1,1	19,0±1,0	13,9±0,6	8,1±8,8			9,9±1,4	19,1± 6,6	7,0±3,0			
**REM%** CG		5 studies	19,2±0,2	18,1±0,5	13,9±1,9	12,1±4,1			13,2±1,6	18,5± 8,4	14,0±2,0			
**REML** SG		4 studies				114,5±74,7		92,0±65,0	91,2±13,5	62,8± 60,2				
**REML** CG		4 studies				136,1±50,0		95,0±62,0	72,0±8,3	81,9± 63,3				
**REMD** SG		1 study							0,09±0,01					
**REMD** CG		1 study							0,09±0,03					
**AI** SG		1 study									64,0±16,0			
**AI** CG		1 study									20,0±3,0			
**Power Spectral Analysis**														
**n_spindles/sigma activity** SG	3 studies							8,4±4,6 (sigma 12,25–16,0 Hz)	68,9±21,6 (n_spindles)	26,1±29,1 (n_spindles)				
**n_spindles/sigma activity** CG	3 studies							10,6±5,4 (sigma 12,25–16,0 Hz)	95,2±30,2 (n_spindles)	39,8±23,5 (n_spindles)				
**Delta 1 (0,75–2,0 Hz)** SG	1 study							185±86						
**Delta 1 (0,75–2,0 Hz)** CG	1 study							183±114						
**Delta 2 (0,75–4,5 Hz)** SG	1 study							230±97						
**Delta 2 (0,75–4,5 Hz)** CG	1 study							239±142						
**Theta (4,75–8,0 Hz)** SG	1 study							29,8±23,0						
**Theta (4,75–8,0 Hz)** CG	1 study							30,2±16,8						
**Alpha (8,25–12,0 Hz)** SG	1 study							17,7±10,8						
**Alpha (8,25–12,0 Hz)** CG	1 study							20,0±11,1						
**Daytime functioning**														
**ESS** SG	2 studies		9,3±0,3											
	2 studies		5,6±0,5											
**Other variables**														
**AHI** SG	4 studies		1,8±0,2	8,9±0,4	30,3±3,3				25,0±7,2			44,0±12,0		
**AHI** CG	4 studies		1,2±0,1	9,0±0,2	33,1±1,8				16,7±3,3			12,0±7,0		
**PLMI** SG	1 study								15,2±4,5					
**PLMI** CG	1 study								9,0±3,0					
**Studies excluded in the meta-analysis (but included in systematic review)**														
**Study**	** **		**Coelho et al 2010 [22]**	**Siccoli et al 2008 [25] ACUTE (means and ranges)**	**Siccoli et al 2008 [25] RECOVERY (means and ranges)**	**Bliwise et al 2002 [23] PARKINSON**	**Bliwise et al 2002 [23] ALZHEIMER**	**Vock et al 2002 [31] ACUTE (means and ranges)**	**Vock et al 2002 [31] MORE CHRONIC (means and ranges)**	**Pinto et al. 2000 [35]**	**Yokoyama et al 1996 [24]**	** **	** **	** **
**Sleep continuity**														
**TST** SG	*/*			395(221–568)	461(406–514)	327,2±95,6	327,2±95,6	390(221–568)	415(302–514)					
**TST** CG	*/*			411(380–451)	411(380–451)	313,3±86,1	255,4±94,8	413(352–493)	413(352–493)					
**SEI** SG	*/*		66,7 ±3,3	81(49–96)	94(88–98)	67,8±20,7	67,8±20,7	76(49–97)	92(63–98)					
**SEI** CG	*/*		69,2 ±3,1	88(80–96)	88(80–96)	70,5±16,7	55,5±23,8	88(71–98)	88(71–98)					
**SOL** SG	*/*			25(3–55)	23(2–45)	16,6±26,4	16,6±26,4	23(10–152)	23(1–51)					
**SOL** CG	*/*			20(17–23)	20(17–23)	31,6±29,6	45,2±70,0	23(8–65)	23(8–65)					
**WASOmin** SG	*/*			91(13–229)	27(5–56)			133(13–229)	29(5–188)					
**WASOmin** CG	*/*			50(9–93)	50(9–93)			52(9–129)	52(9–129)					
**WASO%** SG	*/*													
**WASO%**CG	*/*													
**NA** SG	*/*													
**NA** SG	*/*													
**Sleep architecture**														
**S1min** SG	*/*			50(15–107)	64(13–197)			52(15–105)	52(13–110)					
**S1min** CG	*/*			65(33–117)	65(33–117)			63(20–117)	63(20–117)					
**S1%** SG	*/*													
**S1%** CG	*/*													
**S2min** SG	*/*			282(142–425)	269(200–327)			231(142–425)	253(156–327)					
**S2min** CG	*/*			227(209–253)	227(209–253)			236(191–269)	236(191–269)					
**S2%** SG	*/*													
**S2%** CG	*/*													
**SWSmin** SG	*/*			37(0–141)	36(0–124)			32(0–141)	21(1–94)					
**SWSmin** CG	*/*			35(10–67)	35(10–67)			28(1–89)	28(1–89)					
**SWS%** SG	*/*													
**SWS%** CG	*/*													
**REMmin** SG	*/*			73(14–103)	92(52–149)			69(14–107)	69(32–149)					
**REMmin** CG	*/*			85(67–100)	85(67–100)			88(46–108)	88(46–108)					
**REM%** SG	*/*		12,3 ± 1,5			9,1±6,8	9,1±6,8							
**REM%** CG	*/*		13,4 ± 1,4			17,1±10,0	14,3±11,1							
**REML** SG	*/*					190,2±121,9	190,2±121,9	94(34–479)	99(46–218)					
**REML** CG	*/*					113,6±90,2	75,0±78,9	61(47–201)	61(47–201)					
**REMD** SG	*/*									*				
**REMD** CG	*/*													
**AI** SG	*/*													
**AI** CG	*/*													
**Power Spectral Analysis**														
**PSA**	*/*										results presented graphical#			
**Daytime functioning**														
**ESS** SG	*/*													
**ESS** CG	*/*													
**Other variables**														
**AHI** SG	*/*		14,4 ± 2,7			19,4±22,5(RDI)	19,4±22,5(RDI)							
**AHI** CG	*/*		20,6 ± 4,3			11,7±17,6 (RDI)	19,6±23,5 (RDI)							
**PLMI** SG	*/*		11,7 ± 3,4			41,1±62,3	41,1±62,3							
**PLMI** CG	*/*		1,9 ± 0,7			20,1±23,2	19,7±33,3							
**MSLT** SG	*/*					5,9±4,9	5,9±4,9							
**MSLT** CG	*/*					11,5±6,0	12,1±6,7							

* Data given as median and semi-interquartil interval

#Summarizing (from the abstract): ‘SWS may be associated with dysfunction of the cerebral cortex in stroke patients as well as in normal aged subjects’

SG = Stroke Group; CG = Control Group

#### Sleep continuity

Total sleep time (TST: 9 studies): Across all 9 studies heterogeneity was high (I^2^ = 98.6). TST was significantly shorter in patients with stroke compared to control groups (d = -2.1; SE = 0.9; Z-value = -2.5; p = 0.014). Separate analyses of studies using non-hospitalized [21,30,34] (d = -3.5; SE = 1.6; Z-value = -2.3; p = 0.023) or hospitalized control groups [26–29,32,33] (d = -1.1; SE = 0.5; Z-value = -7.6; p = 0.017) confirmed a significant reduction of TST in patients with stroke compared to both control groups. Heterogeneity remained high in both sub-samples (non-hospitalized: I^2^ = 99.1; hospitalized = 89.1). Separate analysis of studies with longer chronicity [32,33] substantially reduced heterogeneity to I^2^ = 65.3.

Sleep efficiency (SEI– 5 studies): Across all studies, heterogeneity was high (I^2^ = 99.3). SEI was significantly poorer in patients with stroke compared to control groups (d = -4.9; SE = 1.8; Z-value = -2.7; p = 0.006). Separate analyses considering only studies comparing hospitalized control groups (26–29) (d = -2.2; SE = 0.8; Z-value = -2.9; p = 0.004) showed similar results. Heterogeneity remained high (I^2^ = 92.8).

Sleep onset latency (SOL -7 studies): Across all studies, heterogeneity was high (I^2^ = 99.1) and no group differences were evident (d = 1.5; SE = 1.1; Z-value = 1.3; p = 0.188). Separate analyses comparing non-hospitalized [21,30] (d = 3.8; SE = 2.0; Z-value = 1.9; p = 0.055) or hospitalized control groups [26–29,33] (d = -0.3; SE = 0.5; Z-value = -0.6; p = 0.538) showed a marginally significant increase in SOL in patients with stroke as compared to non-hospitalized samples. Heterogeneity remained high in both sub-samples (non-hospitalized: I^2^ = 99.5; hospitalized = 88.4).

Wake after sleep onset (WASO– 5 studies): Three studies reported WASO in minutes [27,28,30], and the other two in percentage of the sleep period time (SPT) [26,33]. Therefore, we conducted 2 separate meta-analyses. In both, heterogeneity was high (WASO_min_: I^2^ = 93.2; WASO_%_: I^2^ = 82.3). Both analyses revealed a trend for greater WASO in stroke than controls (WASO_min_: d = 1.9; SE = 1.0; Z-value = 1.9; p = 0.054; WASO_%_: d = 1.5; SE = 0.8; Z-value = 1.9; p = 0.057). WASO data could not be analysed separately for type of control or stroke chronicity due to lack of data.

#### Sleep architecture

Sleep stages (Stage 1 and 2–6 studies, slow-wave-sleep (SWS) = 7 studies; REM sleep = 8 studies): The duration of sleep stages was reported either in minutes or as percentage of sleep duration measured in reference to the sleep period time (SPT), time in bed (TIB) or TST. This diversity of methods made comparisons difficult. Of the 5 studies expressing the duration of sleep stages as percentages, 3 reported percentages in reference to SPT [26,29,33], while 2 did not specify whether SPT, TIB or TST used [21,30].

For stage 1 sleep (S1), 2 studies reported S1_min_ [27,28], 4 studies reported S1_%_ [26,29,30,33]. For S1_min_, heterogeneity was low (I^2^ = 35.5) and no significant group difference was found (d = -0.1; SE = 0.3; Z-value = -0.2; p = 0.823). For S1_%_, heterogeneity was high (I^2^ = 84.6) and the results showed greater S1_%_ in stroke than the control groups (d = 1.0; SE = 0.5; Z-value = 2.0; p = 0.046).

For stage 2 sleep (S2), 2 studies reported S2_min_ [27,28] The other 4 studies reported S2_%_ [26,29,30,33]. In both meta-analyses, heterogeneity was low or moderate (S2_min_: I^2^ = 0.0; S2_%_: I^2^ = 73.7). For S2_min_, no significant group difference was found (d = 0.1; SE = 0.3; Z-value = 0.3; p = 0.746). S2_%,_ was significantly lower in patients than controls (d = -1.0; SE = 0.4; Z-value = -2.8; p = 0.005).

For SWS, 2 studies reported SWS_min_ [27,28], and 5 studies reported SWS_%_ [21,26,29,30,33]. In both meta-analyses, heterogeneity was moderate or high (SWS_min_: I^2^ = 60.4; SWS_%_: I^2^ = 98.2). SWS_min_ and SWS_%_ were lower in stroke than controls with a trend for SWS_min_ and a significant reduction for SWS_%_ (SWS_min_ = d = -0.8; SE = 0.4; Z-value = -1.8; p = 0.069; SWS_%_ = d = -1.9; SE = 0.8; Z-value = -2.3; p = 0.020). Separate analysis was only possible for SWS_%_ of type of control group. This analysis revealed a significant reduction of SWS_%_ in relation to hospitalised controls (d = -2.0; SE = 0.8; Z-value = -2.6; p = 0.010) but not in comparison to non-hospitalised controls (d = -1.8; SE = 1.2; Z-value = -1.5; p = 0.145).

For REM sleep, 3 studies reported REM_min_ [27,28,32], and 5 studies reported REM_%_ [21,26,29,30,33]. For both parameters, heterogeneity was high (REM_min_: I^2^ = 90.4; REM_%_: I^2^ = 99.3). REM_min_ showed a trend for shorter REM duration for stroke than controls (d = -1.2; SE = 0.7; Z-value = -1.2; p = 0.050), while REM_%_ was insignificant (d = -1.6; SE = 1.3; Z-value = -1.2; p = 0.241). Separate analyses for REM_%_ showed a significant difference between stroke and hospitalised controls [26,29,33] (d = -1.8; SE = 0.7; Z-value = -2.4; p = 0.016), but not non-hospitalized controls [21,30] (d = -1.4; SE = 2.1; Z-value = -0.7; p = 0.515). For REM_min_, no studies with non-hospitalised controls were available.

REM sleep latency (REML– 4 studies): Across all studies [26,28–30] heterogeneity was high (I^2^ = 85.8) and no group difference was found (d = 0.2; SE = 0.5; Z-value = 0.5; p = 0.605). This picture remained when excluding [30] which was the only study using a healthy control group (d = 0.4; SE = 0.6; Z-value = 0.7; p = 0.490).

#### Other sleep variables

Apnea hypopnea index (AHI-3 studies): Three studies reported the AHI [21,34,36]. Heterogeneity was high (I^2^ = 97.2), and no group differences were found (d = 1.1; SE = 1.4; Z-value = 1.0; p = 0.333). However, excluding the study by Arzt et al [21], which comprised three groups of patients and three groups of matched non-hospitalized controls, lead to significantly higher AHI in patients then controls across the remaining two studies [29,34] (d = 2.2; SE = 0.9; z-value = 2.4; p = 0.016).

Number of sleep spindles (2 studies): Two studies reported the number of sleep spindles [29,30]. Heterogeneity was low (I^2^ = 1.6). The number of spindles was significantly lower in the group with stroke compared to controls (d = -0.8; SE = 0.3; Z-value = -3.2; p = 0.001).

REM density, arousal index (AI), periodic limb movement index (PLMI), Epworth Sleepiness Scale (ESS) and multiple sleep latency test (MSLT).

No meta-analysis could be calculated for these variables since only one study reported one of these parameters, respectively. None of the 9 studies qualifying for meta-analyisis reported REM density or MSLT.

## Discussion

This report represents the first systematic review and meta-analysis of studies examining the polysomnographic characteristics of sleep in patients with stroke compared to control populations. This approach allowed us to integrate findings from individual studies with relatively small sample sizes, and hence to establish the strength of the evidence base as well as the trends arising from this evidence base. Specifically, the systematic review component of the study evaluated data from 410 patients with stroke and 1323 controls. The meta-analysis component of the study pooled data from 276 patients with stroke and 1194 controls for the most frequently reported parameter (total sleep time, reported in all 9 studies qualifying for meta-analysis) to 37 patients and 35 controls for the smallest two studies reporting one specific parameter (sleep spindles).

The picture emerging from the meta-analysis indicates poorer sleep efficiency (patients: range 69–79%, mean 75%; vs controls: range 78–88%, mean 84%), shorter total sleep time (patients: range 100–417 mins, mean 309 mins; vs controls: range 113–426 mins, mean 340 mins), and a tendency for more wake after sleep onset in patients with stroke (patients: range 74–112 mins; mean 97 mins; vs controls: range 50–60, mean 54 mins). Moreover, patients spend more time in stage 1 sleep (patients: range 60–66 mins, mean 63 mins; vs controls: range 58–63 mins, mean 60 mins; %: 13 vs 10) and less time in stage 2 sleep (patients: range 223–247 mins, mean 235 mins; vs controls: range 232–243, mean 238 mins; %: 36 vs 45), and have lower percentage of slow wave sleep (patients: range 31–34 mins, mean 33 mins; vs controls: range 39–45 mins, mean 42 mins; %: 10 vs 12)) compared to control populations. However, the group difference in SWS sleep was restricted to the comparison between stroke patients and hospitalized controls; no group difference was found when stroke patients were contrasted with non-hospitalized controls (14% vs 14%). Moreover, no group differences were identified with respect to REM sleep. Of note, a subgroup analysis for different control groups was not possible for S1 and S2 sleep. It is therefore unclear to what extent the respective difference may depend on the type of control population. Taken together, these data suggest that sleep continuity is poorer in patients with stroke, and further indicate subtle changes for sleep architecture but here the findings are not clear enough to draw firm conclusions. Moreover, those differences observed in sleep architecture variables might reflect an indirect effect of group differences in sleep continuity parameters. We therefore propose that the findings reported here are likely to reflect changes in arousal and cortico-thalamo-cortical oscillations. Specifically, we propose that slow oscillations emerging from intact cortical networks serve as an envelope for other brain oscillations such as slow wave activity or sleep spindles. Lesions of the cortex might compromise this process and hence primarily affect sleep continuity. In addition, the psychological impact of stroke might further increase cognitive (psychosocial) arousal, which in turn contributes to poorer sleep continuity as well. In contrast, REM sleep relies on quite small and distinct networks in subcortical regions less likely to be affected by strokes. Of course, this is a speculative account, which can only be examined through future studies combining PSG with structural neuroimaging.

One of the motivations for the present study was the ambition to determine the effect of potentially important variables on PSG parameters, such as age, sex, type of stroke, and in particular chronicity of stroke. The outcome of the literature search, however, revealed clearly that the evidence base presently available is way to limited to address these questions properly. Thus, only a tentative analysis could be conducted for chronicity of stroke and TST by contrasting the early phase vs one month or later after the stroke to controls respectively. These provisional findings suggest that sleep in patients is not only poorer in the early phase post stroke but also in the post acute and chronic phase, thereby corroborating evidence from self-report data [13–15]) with objective PSG data. However, before firm conclusions can be drawn, well-controlled PSG studies on different stages of recovery are needed to fully characterise sleep in persons living with stroke, and to determine the need and opportunity for recovery-promoting and life-enhancing sleep interventions.

A further observation from the present study concerns the exceptionally poor evidence-base with regards to daytime sleepiness. Thus, even though feeling tired and fatigued are frequently reported by patients with stroke e.g. [37,38], only one published study [23] provided data from the multiple sleep latency test (MSLT) in patients with stroke. These data were thereby compared to a clinical control population comprised of patients with Parkinson and Alzheimer diseases. While the findings suggest that stroke patients were sleepier during the day than the clinical control population, the different pathologies of Parkinson/Alzheimer and the medication used to treat these conditions make firm conclusions impossible. The MSLT-derived evidence for greater daytime sleepiness in stroke can therefore only be seen as suggestive evidence affirming observations from other modalities such as wake EEG (e.g. [39]) and questionnaire-based self evaluations.

Taken together, we conclude that patients with stroke experience poor nocturnal sleep primarily due to disrupted sleep continuity. Whether or not patients also have a greater likelihood to fall asleep during the day remains to be examined.

### The role of Obstructive Sleep Apnea (OSA)

Sleep research has made a substantive contribution to our understanding of risk factors for stroke, and the interdependence of stroke re-occurrence and sleep disorder breathing [40]. In this review we deliberately aimed to review the sleep and stroke literature with a wider focus than OSA and hence considered only PSG studies. PSG comprises electroencephalography (EEG), electromyography (EMG), and electrooculography (EOG), and typically also includes respiratory parameters. However, PSG is not required to determine sleep disordered breathing and there is hence a wealth of literature on this condition in patients with stroke. The notion that sleep after stroke is poorly understood does therefore not apply to the specific case of sleep disordered breathing. In contrast, the evidence-base on OSA in stroke is substantive and very strong.

Unfortunately, studies directly contrasting PSG parameters in patients with and without OSA, and/or in relation to controls were not available. We are therefore unable to determine the extent to which the results obtained through this systematic review are driven by differences in sleep disordered breathing between the groups. However, the meta-analysis included 3 PSG studies that also reported mean AHI values. Those studies[21,29,34] covered a relatively similar range of AHI scores in patients (range: 1.8–44.0, mean: 22.0) and controls (range: 1.2–33, mean: 14.4), yet nevertheless reported poorer sleep in stroke patients. We therefore suggest that greater OSA is unlikely to be an exhaustive explanation for the observations obtained through this systematic review. Rather, we propose that poor sleep in patients with stroke is multifactorial in origin, arising from the combination of enhanced vulnerability for OSA as well as neurophysiological (affection of arousal and sleep networks), other physical (e.g. pain, restricted mobility or lower levels of physical activity) and psychological consequences (e.g. depressed mood or psychological adjustment) of stroke.

### Critical evaluation of the evidence base

With the present study, we sought to collate the data on PSG characteristics of sleep in patients with stroke and to generate working hypotheses and directions for future research. We focused on PSG because it not only represents the methodological gold standard in sleep research, but also because it is the only method affording a reliable characterization of sleep, and the differential diagnosis of the underlying causes of poor sleep. The emerging picture clearly suggests that sleep after stroke is poor, with sleep efficiency ratings well in the range of clinically significant insomnia, and substantially reduced TST. The detrimental effects of chronic insomnia on psychological and physical health are well documented e.g. [41,42]. Moreover, studies by [43,44] have further identified short TST a critical factor for many outcomes, including cardiovascular disease. The findings from the present study therefore hand a clear mandate for further research. At the same time, it is also clear that the evidence base is limited and relatively weak. Out of 55 studies relevant to the topic, only 15 included PSG measurements and satisfied our relatively liberal inclusion criteria for systematic review, and only nine of them were suitable for meta-analysis. The 15 studies included were methodologically diverse and weak in particular with regards to control group characteristics and stroke chronicity. For example, some studies used patients with other neurological conditions (Alzheimer, Parkinson or peripheral neurological disorders) rather than healthy participants as controls. The sample characteristics were not always described with sufficient detail, including aspects such as clinical assessment or comorbidities. In addition, the majority of studies focused on the early phase of stroke recovery, while other studies collated data across the acute, sub-acute and/or chronic recovery. The latter is particularly problematic for two reasons. First, changes in sleep early after stroke do not necessarily represent an intrinsic sleep problem, but might be caused by consciousness alterations, medication, or the hospital environment. Second, patients recovering from stroke may experience a plethora of changes on the physiological as well as the psychological level, which are likely to impact their sleep. It is therefore plausible that transient sleep disturbances will go through a process of change over time. Not taking chronicity into consideration might therefore skew the results. Finally, most studies relied on one PSG night only. This is in contrast to the commonly accepted best practice to implement an adaptation night, which is typically discarded for the main analysis.

In our view, the critical assessment of the literature, as discussed above, suggests that more PSG research comparing stroke to healthy controls is needed to strengthen and expand the existing evidence base. Importantly, large-scale methodologically stringent studies controlling for type of stroke and chronicity as well as selection bias, and including healthy age- and gender-matched controls, are needed. Such well-controlled studies are difficult and costly to pursue which probably explains the relatively limited evidence-base available at present. This may be best overcome through multi-centre trials, and further be aided by new technology affording PSG recordings in the home. The collated evidence obtained through this systematic review is, in our view, an important step in this direction. Moreover, because of the known interaction of sleep and daytime function, the evidence reported here holds wider implications for stroke rehabilitation and recovery. These considerations and their clinical relevance are explored further below.

### Implications for stroke care

Sleep is known to be important for health and well-being in all persons, but increasingly so in those recovering from brain damage and those sustaining chronic deficits after stroke. For example, healthy restorative sleep, and especially deep sleep, which the meta-analysis suggests to be reduced in patients, is known to promote neural plasticity, learning, and memory consolidation [45,46]. These plasticity-promoting characteristics of sleep are likely to be particularly relevant in situations requiring substantive reorganization and re-learning, such as neurorehabilitation. Moreover, many patients with stroke suffer from depression [47]. At the same time, depression is also strongly associated with poor sleep [48]. A better understanding of sleep after stroke is therefore not only relevant for rehabilitation and long-term outcome, but also for mental health and quality of life. In contrast to its importance, at present sleep is often not adequately considered in stroke care, except for sleep-disordered breathing. In our view, this position should be extended to a more holistic approach to stroke care provision by including the diagnosis and treatment of sleep problems, as well as the performance of intervention studies to determine whether sleep-related interventions might enhance rehabilitation efforts.

**Fig 3:**
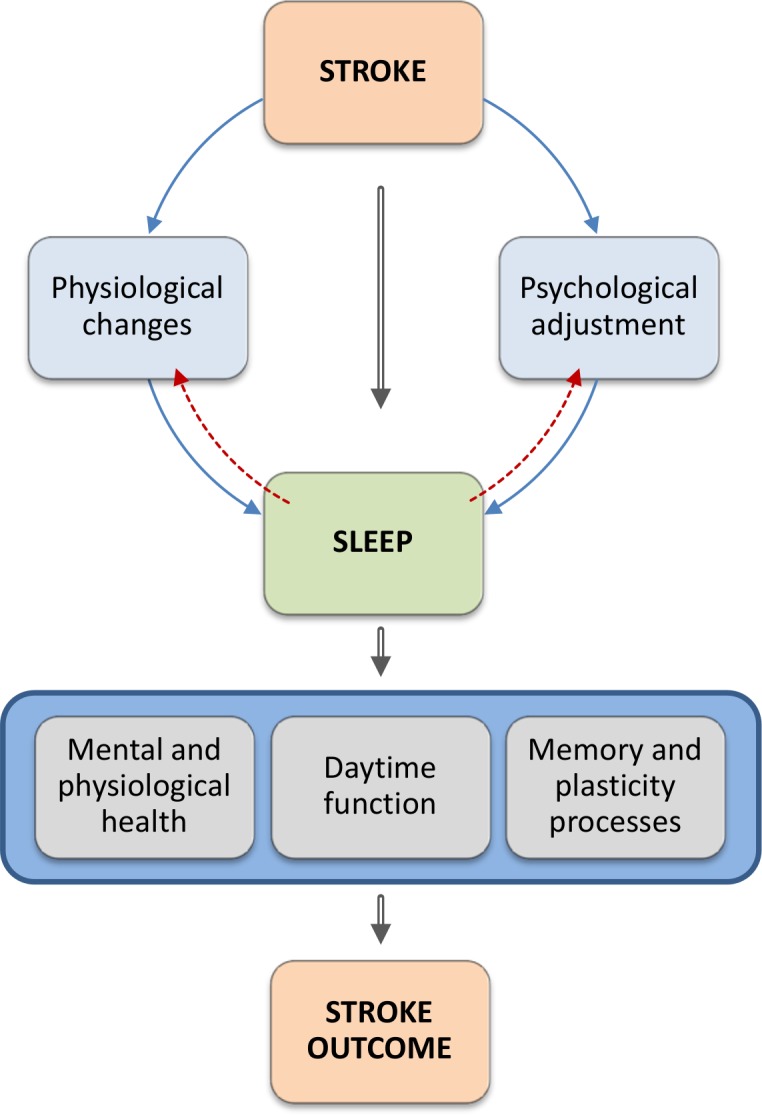
Schematic representation of the how sleep changes in stroke might interact with stroke recovery. Having a stroke induces physiological and psychological changes that are likely to trigger sleep problems. Sleep on the other hand is associated with mental and physical health, influences memory and plasticity processes, and is an important mediator of daytime functioning and performance. It is therefore plausible to assume that poor sleep impacts negatively on all of these aspects and may therefore directly or indirectly be detrimental to the rehabilitation process and longterm outcome.

A further important question relates to the etiology of sleep difficulties after stroke, and the possibility of sleep disorders progressing from a secondary symptom of stroke to a primary sleep disorder co-morbid to the stroke. As illustrated in Fig 3, we propose a mechanism which might lead to this situation. Thus, we propose that physiological and psychological changes, induced by the stroke event and treatment thereof, are likely to trigger poor sleep in the early phase of stroke recovery. Based on Spielman’s 3P model of insomnia [49], which suggests the presence of predisposing, precipitating, and perpetuating factors playing a role in the pathophysiology of the disorder, we assume that in some cases sleep problems may be maintained into the chronic phase through psychological processes such as maladaptive behaviors and dysfunctional beliefs, as well as physiological aspects including lesion characteristics and medication. At the same time, as a consequence of poor sleep, mood and motivation might be reduced, and patients are less likely to be as active as they can be, and to fully engage in rehabilitation activities. This in turn reduces the stimulation necessary to drive functional reorganization and also limits the opportunity for sleep to induce memory consolidation. As a result, functional recovery and long-term outcome may be compromised. Moreover, daytime health behaviors such as sedentary life style, high levels of inactivity, and extensive napping might further aggravate difficulties with sleep maintenance and continuity, thereby causing a vicious cycle. A better understanding of sleep in stroke survivors, and the physiological and psychological factors contributing these sleep difficulties is therefore needed for two reasons, to prevent chronic comorbid sleep disorders and to maximize the efficacy of neurological rehabilitation.

## Conclusions

The present study draws together the existing literature on sleep polysomnography in stroke compared to controls for the first time. Although the number of studies is small, and methodologically diverse, they clearly identify sleep as a problem in patients with stroke, and highlight the need to give greater consideration to sleep in stroke care, and in particular in stroke rehabilitation. At present, sleep is not routinely considered in stroke care. This is reflected e.g. by the new guidelines on stroke rehabilitation, published by the National Institute for Health and Care Excellence in the UK which make no reference to sleep or sleep disordered breathing. This is most likely due to the scarcity of research and relative weakness of the evidence base. How sleep is affected by stroke and how stroke outcome may be affected by sleep is yet to be determined. The latter will require a concerted, ideally multi-centered program of research to enable the determination of critical modulators of the sleep-recovery interaction, such as type of stroke and lesion characteristics.

## Supporting Information

S1 PRISMA Checklist PRISMA Checklist. (DOC)

S1 Table Definition of sleep variables. (DOC)

S2 Table List of studies failing the including criteria for systematic review/meta-analysis. (DOCX)
