# Correction: Polysomnographic Characteristics of Sleep in Stroke: A Systematic Review and Meta-Analysis

**DOI:** 10.1371/journal.pone.0155652

**Published:** 2016-05-11

**Authors:** Chiara Baglioni, Christoph Nissen, Adrian Schweinoch, Dieter Riemann, Kai Spiegelhalder, Mathias Berger, Cornelius Weiller, Annette Sterr

The following information is missing from the Funding section: The article processing charge was funded by the German Research Foundation (DFG) and the Albert Ludwigs University Freiburg in the funding programme Open Access Publishing.
