# Supplementary material for: Polysomnographic Characteristics of Sleep in Stroke: A Systematic Review and Meta-Analysis
Source: PLoS One. 2016 Mar 7;11(3):e0148496. doi: 10.1371/journal.pone.0148496 (PMC4780740; doi:10.1371/journal.pone.0148496)
Supplement: S1 Table — (DOC) [file pone.0148496.s002.doc]

| **Sleep variable** | **Abbreviation** | **Description** |
| --- | --- | --- |
| Total Sleep Time | TST | The total time spent asleep during the recording night |
| Sleep Efficiency Index | SEI | Ratio of TST to time in bed x 100 % |
| Sleep Onset Latency | SOL | Time from lights out until sleep onset (defined as first epoch of stage 2) |
| Wake After Sleep Onset | WASO | The difference between sleep period time (SPT; time from sleep until final awakening) and TST |
| Number of Awakenings | NA | The total number of awakenings during the night |
| Percentages of Stage 1, Stage 2, Slow Wave Sleep, and REM Sleep | S1, S2, SWS, REM | Amount of each stage as percentage (generally of SPT) |
| REM sleep Latency | REM L | After a person falls asleep, the amount of time it takes for the first onset of REM sleep |
| REM sleep Density | REM D | An index which represents the frequency of rapid eye movements during REM sleep |
| Arousal Index | AI | The number of arousals (abrupt change from sleep to wakefulness, or from a "deeper" stage of non-REM sleep to a "lighter" stage) per hour |
| Sleep Spindles | SS | Powerful bursts of oscillations in the 11–16 Hz frequency range, lasting from 0.5–3s and arising from thalamocortical circuitries, which are prevalent in stage 2 sleep and have been correlated with memory processes |
| Epworth Sleepiness Scale | ESS | A short questionnaire intended to measure daytime sleepiness. The score ranges between 0 and 24, with higher scores indicating more sleepiness |
| Power spectral analyses | PSA | A method for quantifying the EEG's constituting frequency components (e.g. sigma activity/spindles), theta including those that reflect the general arousal level of the brain |
| Apnea-hypopnea index | AHI | An index used to assess the severity of [sleep apnea](http://sleepdisorders.about.com/od/commonsleepdisorders/a/Sleep-Apnea-Overview.htm) based on the total number of complete cessations (apnea) and partial obstructions (hypopnea) of breathing occurring per hour of sleep. |
| Periodic Limb Movement Index | PLMI | The number of periodic limb  movements per hour of sleep |
| Multiple Sleep Latency Test | MSLT | A method which measures the strength of daytime sleep tendency. It measures the speed of falling asleep in a regulated environment at five times during the day, at two hour intervals |

Table S1: Definition of the considered sleep variables
