# Supplementary material for: Polysomnographic Characteristics of Sleep in Stroke: A Systematic Review and Meta-Analysis
Source: PLoS One. 2016 Mar 7;11(3):e0148496. doi: 10.1371/journal.pone.0148496 (PMC4780740; doi:10.1371/journal.pone.0148496)
Supplement: S2 Table — (DOCX) [file pone.0148496.s003.docx]

**Table S2: List of the 40 excluded identified records**

| **Study** | **No control group** | **No PSG** | **Mixed patients group (mixed disorders)** | **Case- reports** | **Brain injury other** |
| --- | --- | --- | --- | --- | --- |
| Bassetti et al.; 2006 (1) | X |  |  |  |  |
| Bassetti and Aldrich.; 1999a [(2)](#_ENREF_2) | X |  |  |  |  |
| Bassetti and Aldrich; 1999b [(3)](#_ENREF_3) |  | X (sleep apnea) |  |  |  |
| Bassetti et al.; 1996 [(4)](#_ENREF_4) |  | X (AHI) |  |  |  |
| Bravata et al.; 2010 [(5)](#_ENREF_5) | X |  |  |  |  |
| Cadilhac et al.; 2005 [(6)](#_ENREF_6) | X |  |  |  |  |
| Chami et al.; 2011 [(7)](#_ENREF_7) |  |  | X (myocardial infarction, congestive heart failure, and stroke) |  |  |
| Dyken et al.; 1996 (8) |  | X (AHI) |  |  |  |
| Dziewas et al.; 2005 (9) | X |  |  |  |  |
| Dziewas et al.; 2008 (10) | X |  |  |  |  |
| Fang et al.; 2009 (11) | X |  |  |  |  |
| Giubilei et al.; 1998 [(12)](#_ENREF_11) |  | X (heart frequency) |  |  |  |
| Harbison et al.; 2002 (13) | X |  |  |  |  |
| Hsu et al.; 2006  [(14)](#_ENREF_13) | X |  |  |  |  |
| Hui et al.; 2002 [(15)](#_ENREF_14) |  | X (AHI) |  |  |  |
| Iranzo et al.; 2002 [(16)](#_ENREF_15) | X |  |  |  |  |
| Joo et al. 2011 (17) |  | X (AHI) |  |  |  |
| Kaneko et al.; 2003[^17^](#_ENREF_16) | X |  |  |  |  |
| Kario et al.; 2004 (18) |  |  |  | X |  |
| Körner et al.; 1986 (19) | X |  |  |  |  |
| Lawrence et al.; 2001 (20) | X |  |  |  |  |
| Martinez- Garcia et al.; 2004 (21) | X |  |  |  |  |
| Martinez- Garcia et al.; 2005 (22) | X |  |  |  |  |
| Martinez- Garcia et al.; 2009 (23) | X |  |  |  |  |
| McArdle et al., 2003 (24) |  |  |  |  | X |
| Parra et al.; 2011 (25) | X |  |  |  |  |
| Parra et al.; 2004 (26) | X |  |  |  |  |
| Parra et al.; 2000 [(27)](#_ENREF_25) |  | X (AHI) |  |  |  |
| Roffe et al.; 2003 (28) |  | X (oximetry) |  |  |  |
| Roffe et al.; 2010 [(29)](#_ENREF_28) |  | X (oxygen saturation) |  |  |  |
| Rola et al.; 2008 [(30)](#_ENREF_29) | X |  |  |  |  |
| Rola et al.; 2007 [(31)](#_ENREF_30) |  | X (AHI) |  |  |  |
| Ron et al.; 1980 (32) |  |  |  |  | X ( Traumatic brain- injuries) |
| Sahlin et al.; 2008 (33) | X |  |  |  |  |
| Sandberg et al.; 2001 (34) | X |  |  |  |  |
| Svatikova et al.; 2011 [(35)](#_ENREF_34) | X |  |  |  |  |
| Szucs et al.; 2002 [(36)](#_ENREF_35) |  | X (oxygen desaturation index) |  |  |  |
| Turkington et al.; 2004 [(37)](#_ENREF_36) | X |  |  |  |  |
| Wessendorf et al.; 2001 [(38)](#_ENREF_37) | X |  |  |  |  |
| Wierzbicka et al.; 2006  [(39)](#_ENREF_38) | X |  |  |  |  |

References

1. Bassetti CL, Milanova M, Gugger M. Sleep-disordered breathing and acute ischemic stroke: diagnosis, risk factors, treatment, evolution, and long-term clinical outcome. Stroke [Internet]. 2006/03/18 ed. 2006;37(4):967–72. Available from: http://www.ncbi.nlm.nih.gov/pubmed/16543515

2. Bassetti C, Aldrich MS. Sleep apnea in acute cerebrovascular diseases: final report on 128 patients. Sleep [Internet]. 1999/04/14 ed. 1999;22(2):217–23. Available from: http://www.ncbi.nlm.nih.gov/pubmed/10201066

3. Bassetti C, Aldrich M. Night time versus daytime transient ischaemic attack and ischaemic stroke: a prospective study of 110 patients. J Neurol Neurosurg Psychiatry [Internet]. 1999/09/16 ed. 1999;67(4):463–7. Available from: http://www.ncbi.nlm.nih.gov/pubmed/10486392

4. Bassetti C, Mathis J, Gugger M, Lovblad KO, Hess CW. Hypersomnia following paramedian thalamic stroke: a report of 12 patients. Ann Neurol [Internet]. 1996/04/01 ed. 1996;39(4):471–80. Available from: http://www.ncbi.nlm.nih.gov/pubmed/8619525

5. Bravata DM, Concato J, Fried T, Ranjbar N, Sadarangani T, McClain V, et al. Auto-titrating continuous positive airway pressure for patients with acute transient ischemic attack: a randomized feasibility trial. Stroke [Internet]. 2010/05/29 ed. 2010;41(7):1464–70. Available from: http://www.ncbi.nlm.nih.gov/pubmed/20508184

6. Cadilhac DA, Thorpe RD, Pearce DC, Barnes M, Rochford PD, Tarquinio N, et al. Sleep disordered breathing in chronic stroke survivors. A study of the long term follow-up of the SCOPES cohort using home based polysomnography. J Clin Neurosci. 2005;12(6):632–7.

7. Chami HA, Resnick HE, Quan SF, Gottlieb DJ. Association of incident cardiovascular disease with progression of sleep-disordered breathing. Circulation [Internet]. 2011/03/16 ed. 2011;123(12):1280–6. Available from: http://www.ncbi.nlm.nih.gov/pubmed/21403097

8. Dyken ME, Somers VK, Yamada T, Ren ZY, Zimmerman MB. Investigating the relationship between stroke and obstructive sleep apnea. Stroke [Internet]. 1996/03/01 ed. 1996;27(3):401–7. Available from: http://www.ncbi.nlm.nih.gov/pubmed/8610303

9. Dziewas R, Hopmann B, Humpert M, Bontert M, Dittrich R, Ludemann P, et al. Capnography screening for sleep apnea in patients with acute stroke. Neurol Res [Internet]. 2005/04/15 ed. 2005;27(1):83–7. Available from: http://www.ncbi.nlm.nih.gov/pubmed/15829165

10. Dziewas R, Hopmann B, Humpert M, Ritter M, Dittrich R, Schabitz WR, et al. Positional sleep apnea in patients with ischemic stroke. Neurol Res [Internet]. 2008/04/22 ed. 2008;30(6):645–8. Available from: http://www.ncbi.nlm.nih.gov/pubmed/18423112

11. Fang Y, Li Y. [One stage multilevel surgery for patients with moderate and severe obstructive sleep apnea hypopnea syndrome]. Zhonghua Er Bi Yan Hou Tou Jing Wai Ke Za Zhi [Internet]. 2009/12/08 ed. 2009;44(8):687–9. Available from: http://www.ncbi.nlm.nih.gov/pubmed/19961779

12. Giubilei F, Strano S, Lino S, Calcagnini G, Tisei P, Fiorelli M, et al. Autonomic nervous activity during sleep in middle cerebral artery infarction. Cerebrovasc Dis [Internet]. 1998/04/21 ed. 1998;8(2):118–23. Available from: http://www.ncbi.nlm.nih.gov/pubmed/9548011

13. Harbison J, Ford GA, James OF, Gibson GJ. Sleep-disordered breathing following acute stroke. QJM [Internet]. 2002/10/23 ed. 2002;95(11):741–7. Available from: http://www.ncbi.nlm.nih.gov/pubmed/12391386

14. Hsu CY, Vennelle M, Li HY, Engleman HM, Dennis MS, Douglas NJ. Sleep-disordered breathing after stroke: a randomised controlled trial of continuous positive airway pressure. J Neurol Neurosurg Psychiatry [Internet]. 2006/06/15 ed. 2006;77(10):1143–9. Available from: http://www.ncbi.nlm.nih.gov/pubmed/16772358

15. Hui DS, Choy DK, Wong LK, Ko FW, Li TS, Woo J, et al. Prevalence of sleep-disordered breathing and continuous positive airway pressure compliance: results in chinese patients with first-ever ischemic stroke. Chest [Internet]. 2002/09/13 ed. 2002;122(3):852–60. Available from: http://www.ncbi.nlm.nih.gov/pubmed/12226023

16. Iranzo A, Santamaria J, Berenguer J, Sanchez M, Chamorro A. Prevalence and clinical importance of sleep apnea in the first night after cerebral infarction. Neurology [Internet]. 2002/03/27 ed. 2002;58(6):911–6. Available from: http://www.ncbi.nlm.nih.gov/pubmed/11914407

17. Joo BE, Seok HY, Yu SW, Kim BJ, Park KW, Lee DH, et al. Prevalence of sleep-disordered breathing in acute ischemic stroke as determined using a portable sleep apnea monitoring device in Korean subjects. Sleep Breath [Internet]. 2010/01/23 ed. 2011;15(1):77–82. Available from: http://www.ncbi.nlm.nih.gov/pubmed/20094807

18. Kario K, Morinari M, Murata M, Katsuki T, Shimada K. Nocturnal onset ischemic stroke provoked by sleep-disordered breathing advanced with congestive heart failure. Am J Hypertens [Internet]. 2004/07/06 ed. 2004;17(7):636–7. Available from: http://www.ncbi.nlm.nih.gov/pubmed/15233984

19. Korner E, Flooh E, Reinhart B, Wolf R, Ott E, Krenn W, et al. Sleep alterations in ischemic stroke. Eur Neurol [Internet]. 1986/01/01 ed. 1986;25 Suppl 2:104–10. Available from: http://www.ncbi.nlm.nih.gov/pubmed/3758112

20. Lawrence E, Dundas R, Higgens S, Howard R, Rudd A, Wolfe C, et al. The natural history and associations of sleep disordered breathing in first ever stroke. Int J Clin Pract [Internet]. 2002/01/05 ed. 2001;55(9):584–8. Available from: http://www.ncbi.nlm.nih.gov/pubmed/11770352

21. Martínez García MA, Galiano Blancart R, Cabero Salt L, Soler Cataluña JJ, Escamilla T, Román Sánchezc P. Prevalence of Sleep-Disordered Breathing in Patients With Acute Ischemic Stroke: Influence of Onset Time of Stroke. Arch Bronconeumol. 2004;40(5):196–202.

22. Martínez García MA, Galiano Blancart R, Román Sánchezc P, Soler Cataluña JJ, Cabero Salt L, Salcedo Maiques E. Continuous Positive Airway Pressure Treatment in Sleep Apnea Prevents New Vascular Events After Ischemic Stroke. Chest. 2005;128(4):2123–9.

23. Martínez García MA, Soler Cataluña JJ, Ejarque Martínez L, Soriano Y, Román Sánchezc P, Barbe Illa F, et al. Continuous Positive Airway Pressure Treatment Reduces Mortality in Patients with Ischemic Stroke and Obstructive Sleep Apnea A 5-Year Follow-up Study. Am J Respir Crit Care Med. 2009;180:36–41.

24. McArdle N, Riha RL, Vennelle M, Coleman EL, Dennis MS, Warlow CP, et al. Sleep-disordered breathing as a risk factor for cerebrovascular disease: a case-control study in patients with transient ischemic attacks. Stroke [Internet]. 2003/12/06 ed. 2003;34(12):2916–21. Available from: http://www.ncbi.nlm.nih.gov/pubmed/14657548

25. Parra O, Sanchez-Armengol A, Bonnin M, Arboix A, Campos-Rodriguez F, Perez-Ronchel J, et al. Early treatment of obstructive apnoea and stroke outcome: a randomised controlled trial. Eur Respir J [Internet]. 2010/09/18 ed. 2011;37(5):1128–36. Available from: http://www.ncbi.nlm.nih.gov/pubmed/20847081

26. Parra O, Arboix A, Montserrat JM, Quinto L, Bechich S, Garcia-Eroles L. Sleep-related breathing disorders: impact on mortality of cerebrovascular disease. Eur Respir J [Internet]. 2004/08/31 ed. 2004;24(2):267–72. Available from: http://www.ncbi.nlm.nih.gov/pubmed/15332396

27. Parra O, Arboix A, Bechich S, Garcia-Eroles L, Montserrat JM, Lopez JA, et al. Time course of sleep-related breathing disorders in first-ever stroke or transient ischemic attack. Am J Respir Crit Care Med [Internet]. 2000/02/15 ed. 2000;161(2 Pt 1):375–80. Available from: http://www.ncbi.nlm.nih.gov/pubmed/10673174

28. Roffe C, Sills S, Halim M, Wilde K, Allen MB, Jones PW, et al. Unexpected nocturnal hypoxia in patients with acute stroke. Stroke [Internet]. 2003/10/25 ed. 2003;34(11):2641–5. Available from: http://www.ncbi.nlm.nih.gov/pubmed/14576377

29. Roffe C, Frohnhofen H, Sills S, Hodsoll J, Allen MB, Jones PW. Frequency of nocturnal hypoxia in clinically stable patients during stroke rehabilitation. Clin Rehabil [Internet]. 2010/02/17 ed. 2010;24(3):267–75. Available from: http://www.ncbi.nlm.nih.gov/pubmed/20156982

30. Rola R, Jarosz H, Wierzbicka A, Wichniak A, Richter P, Ryglewicz D, et al. Sleep disorderd breathing and recurrence of cerebrovascular events, case-fatality, and functional outcome in patients with ischemic stroke or transient ischemic attack. J Physiol Pharmacol [Internet]. 2009/02/28 ed. 2008;59 Suppl 6:615–21. Available from: http://www.ncbi.nlm.nih.gov/pubmed/19218688

31. Rola R, Wierzbicka A, Wichniak A, Jernajczyk W, Richter P, Ryglewicz D. Sleep related breathing disorders in patients with ischemic stroke and transient ischemic attacks: respiratory and clinical correlations. J Physiol Pharmacol [Internet]. 2008/03/28 ed. 2007;58 Suppl 5(Pt 2):575–82. Available from: http://www.ncbi.nlm.nih.gov/pubmed/18204171

32. Ron S, Algom D, Hary D, Cohen M. Time-related changes in the distribution of sleep stages in brain injured patients. Electroencephalogr Clin Neurophysiol [Internet]. 1980/04/01 ed. 1980;48(4):432–41. Available from: http://www.ncbi.nlm.nih.gov/pubmed/6153604

33. Sahlin C, Sandberg O, Gustafson Y, Bucht G, Carlberg B, Stenlund H, et al. Obstructive sleep apnea is a risk factor for death in patients with stroke: a 10-year follow-up. Arch Intern Med [Internet]. 2008/02/13 ed. 2008;168(3):297–301. Available from: http://www.ncbi.nlm.nih.gov/pubmed/18268171

34. Sandberg O, Franklin KA, Bucht G, Gustafson Y. Sleep apnea, delirium, depressed mood, cognition, and ADL ability after stroke. J Am Geriatr Soc [Internet]. 2001/05/12 ed. 2001;49(4):391–7. Available from: http://www.ncbi.nlm.nih.gov/pubmed/11347781

35. Svatikova A, Jain R, Chervin RD, Hagan PG, Brown DL. Echocardiographic findings in ischemic stroke patients with obstructive sleep apnea. Sleep Med [Internet]. 2011/06/22 ed. 2011;12(7):700–3. Available from: http://www.ncbi.nlm.nih.gov/pubmed/21689982

36. Szucs A, Vitrai J, Janszky J, Migleczi G, Bodizs R, Halasz P, et al. Pathological sleep apnoea frequency remains permanent in ischaemic stroke and it is transient in haemorrhagic stroke. Eur Neurol [Internet]. 2002/01/23 ed. 2002;47(1):15–9. Available from: http://www.ncbi.nlm.nih.gov/pubmed/11803187

37. Turkington PM, Elliott MW. Sleep disordered breathing following stroke. Monaldi Arch Chest Dis [Internet]. 2005/02/01 ed. 2004;61(3):157–61. Available from: http://www.ncbi.nlm.nih.gov/pubmed/15679009

38. Wessendorf TE, Wang YM, Thilmann AF, Sorgenfrei U, Konietzko N, Teschler H. Treatment of obstructive sleep apnoea with nasal continuous positive airway pressure in stroke. Eur Respir J [Internet]. 2001/11/22 ed. 2001;18(4):623–9. Available from: http://www.ncbi.nlm.nih.gov/pubmed/11716165

39. Wierzbicka A, Rola R, Wichniak A, Richter P, Ryglewicz D, Jernajczyk W. The incidence of sleep apnea in patients with stroke or transient ischemic attack. J Physiol Pharmacol [Internet]. 2006/10/31 ed. 2006;57 Suppl 4:385–90. Available from: http://www.ncbi.nlm.nih.gov/pubmed/17072068
